# Repaglinide Silences the FOXO3/Lumican Axis and Represses the Associated Metastatic Potential of Neuronal Cancer Cells

**DOI:** 10.3390/cells9010001

**Published:** 2019-12-18

**Authors:** Stefan Salcher, Gilles Spoden, Julia M. Huber, Georg Golderer, Herbert Lindner, Michael J. Ausserlechner, Ursula Kiechl-Kohlendorfer, Kathrin Geiger, Petra Obexer

**Affiliations:** 1 Tyrolean Cancer Research Institute, 6020 Innsbruck, Austria; stefan.salcher@tkfi.at (S.S.); gilles.spoden@gmx.net (G.S.); jm.huber@gmx.at (J.M.H.); kathrin.geiger@vivit.at (K.G.); 2 Division of Biological Chemistry, Biocenter, Medical University Innsbruck, 6020 Innsbruck, Austria; georg.golderer@i-med.ac.at; 3 Division of Clinical Biochemistry, Medical University Innsbruck, 6020 Innsbruck, Austria; herbert.lindner@i-med.ac.at; 4 Department of Pediatrics I, Medical University Innsbruck, 6020 Innsbruck, Austria; michael.j.ausserlechner@i-med.ac.at; 5 Department of Pediatrics II, Medical University Innsbruck, 6020 Innsbruck, Austria; ursula.kohlendorfer@i-med.ac.at

**Keywords:** repaglinide, FOXO3, lumican, neuroblastoma, migration

## Abstract

The transcription factor FOXO3 is associated with poor outcome in high-stage neuroblastoma (NB), as it facilitates chemoprotection and tumor angiogenesis. In other tumor entities, FOXO3 stimulates metastasis formation, one of the biggest challenges in the treatment of aggressive NB. However, the impact of FOXO3 on the metastatic potential of neuronal tumor cells remains largely unknown. In the present study, we uncover the small leucine-rich proteoglycan family member lumican (LUM) as a FOXO3-regulated gene that stimulates cellular migration in NB. By a drug-library screen we identified the small molecular weight compound repaglinide (RPG) as a putative FOXO3 inhibitor. Here, we verify that RPG binds to the FOXO3-DNA-binding-domain (DBD) and thereby silences the transcriptional activity of FOXO3. Consistent with the concept that the FOXO3/LUM axis enhances the migratory capacity of aggressive NB cells, we demonstrate that stable knockdown of LUM abrogates the FOXO3-mediated increase in cellular migration. Importantly, FOXO3 inhibition by RPG represses the binding of FOXO3 to the LUM promoter, inhibits FOXO3-mediated LUM RNA and protein expression, and efficiently abrogates FOXO3-triggered cellular “wound healing” as well as spheroid-based 3D-migration. Thus, silencing the FOXO3/LUM axis by the FDA-approved compound RPG represents a promising strategy for novel therapeutic interventions in NB and other FOXO3-dependent tumors.

## 1. Introduction

Neuroblastoma (NB) is a pediatric tumor of the sympathetic nervous system that covers a broad spectrum of clinical outcomes. In high-stage NB, therapy-failure due to acquired chemoresistance and metastasis formation results in a high relapse rate and a five year survival probability of less than 40% [1]. The transcription factor FOXO3, a member of the forkhead box O (FOXO) superfamily, has been associated with elevated tumorigenicity in different cancers [2,3,4,5,6,7,8,9,10]. In concordance, our own studies indicate that active FOXO3 promotes tumor angiogenesis in vivo [11] and chemoprotection in vitro [12] in high-stage NB. Besides its chemoprotective properties [12], several studies point to a supportive role of FOXO3 in facilitating and stimulating metastasis (reviewed in [13]), one of the biggest challenges in the treatment of aggressive NB [1].

Recently, Rehman et al. demonstrated that FOXO3 promotes tumor cell migration and may serve as a prognostic biomarker and a potential therapeutic target for breast cancer [14]. In concordance, Storz et al. found that FOXO3 promotes breast cancer cell invasion through the induction of matrix metalloproteinases 9 and 13 (MMP-9 and MMP-13) [4]. FOXO3 has further been associated with MMP-9 activity and elevated invasion capacity in glioma [15], with increased cell migration and invasion in gastric [16] and colorectal cancers [3]. In non-small cell lung cancer, FOXO3 decreases the expression of the metastasis suppressor gene *nm23-H1* [17]. In line with this, FOXO3-knockdown attenuates tumor growth and metastasis formation in pancreatic ductal carcinoma and in glioblastoma xenografts [8,9]. However, the impact of FOXO3 on the metastatic potential of NB cells remains largely unknown.

Small leucine-rich proteoglycans (SLRPs) are important regulators of extracellular matrix assembly and MMP activity. The SLRP family member lumican (LUM) has been described to both positively and negatively regulate the metastatic potential of different cancers (reviewed in [18]). LUM contributes to the tumorigenesis and metastasis of gastric cancer by activating integrin β1-FAK signaling [19] and its expression correlates with the invasive potential demonstrated in gastric cancer patient samples [20]. In colon cancer, LUM triggers cytoskeletal remodeling and elevates the cellular migration capacity [21], and, in bladder cancer, LUM expression promotes cell proliferation and migration [22]. In glioblastoma and NB, LUM expression is associated with the maintenance of a quiescent, drug-resistant, stem-cell-like phenotype [23]. Here, we report for the first time that LUM is a FOXO3-regulated gene involved in the cellular migration of neuronal tumor cells.

By screening the Prestwick Chemical Library^®^, containing 1120 FDA-approved drugs, we recently identified and characterized carbenoxolone (CBX) as the first FOXO3 inhibitor that overcomes FOXO3-mediated chemoprotection in high-stage NB [24]. In this drug-screen, repaglinide (RPG), an insulin secretagogue belonging to the meglitinide class, was also identified as a putative FOXO3 inhibitory compound [24]. Hence, the present study was designed to investigate the efficacy of RPG to silence the FOXO3/LUM axis and to repress the associated metastatic potential of neuronal cancer cells.

## 2. Materials and Methods

### 2.1. Cell Lines, Culture Conditions, and Reagents

The NB cell line SH-EP was obtained from N. Gross, Lausanne, Switzerland [25] and the NB cell lines SK-N-SH and IMR32 were purchased from ATCC (Rockville, MD, USA). For all cell culture experiments with these cells, RPMI1640 medium (Lonza, Basel, Switzerland) supplemented with 10% fetal calf serum (Sigma-Aldrich, Vienna, Austria), 100 U/mL penicillin, 100 µg/mL streptomycin, and 2 mM L-glutamine (Lonza, Basel, Switzerland) was used. Phoenix^TM^ [26] and HEK293T packaging cells were cultivated in DMEM medium (Lonza, Basel, Switzerland). Using the VenorRGeM-mycoplasma detection kit (Minerva Biolabs, Berlin, Germany), all cells were routinely tested for mycoplasma contamination. All reagents were purchased at Sigma-Aldrich (Vienna, Austria) unless stated otherwise.

### 2.2. Retroviral and Lentiviral Expression Vectors

The retroviral plasmid pLIB-FOXO3(A3)-ER-iresNeo has been described [27]. The vector for human LUM-specific shRNA (sc-43901-SH) was purchased at Santa Cruz Biotechnology (Dallas, TX, USA).

### 2.3. Production of Lentiviruses and Retroviruses for Infection

The generation of lentiviruses and retroviruses has been previously described [28]. SK-N-SH cells were infected with the supernatants of the pLIB-FOXO3(A3)-ER-iresNeo retrovirus to generate SK-N-SH/FOXO3 cells (Figure S1). SH-EP/FOXO3 and IMR32/FOXO3 cells have previously been described [27,29]. SK-N-SH/FOXO3 and IMR32/FOXO3 cells were infected with the scrambled shCTR and the shLUM lentivirus-supernatants to generate SK-N-SH/FOXO3-shCTR, SK-N-SH/FOXO3-shLUM, as well as IMR32/FOXO3-shCTR and IMR32/FOXO3-shLUM cells, respectively.

### 2.4. Generation and Purification of Recombinant FOXO3-DNA-Binding-Domain (DBD) Protein

The generation and the purification of the codon-usage optimized human FOXO3-DBD (residues 156−269) has been previously described [24].

### 2.5. Fluorescence Polarization Assay (FPA)

To analyze the interaction of the substance RPG with the FOXO3-DBD protein, a FPA was performed as described previously [24]. To determine the specificity of RPG a FPA with recombinant 14-3-3 sigma protein and the R18 peptide was conducted as described previously [24]. 

### 2.6. Determination of the Equilibrium Dissociation Constant (Kd), IC_50_, and Binding Affinity (Ki) Value

By FPA the dissociation constant (Kd) for the FOXO3-DBD/IRE-FAM interaction was analyzed as described previously [24]. For determination of the IC_50_-value, 1 µM to 200 µM RPG were incubated with 25 nM FOXO3-DBD and 5 nM IRE-oligonucleotide and analyses of the Ki-value was performed by the equation of Nikolovska-Coleska [30] as described [24].

### 2.7. Fluorescence-Based Electrophoretic Mobility Shift Assay (FAM-EMSA)

The FAM-EMSA and the cell-based FAM-EMSA, with cell extracts of SH-EP/FOXO3 cells, were performed as described previously [24] using fluorescence-labeled, double-stranded 100 nM FoxP3 oligonucleotides (FoxP3-forward: AGCAAAGTTGTTTTTGATAATG, FoxP3-reverse: CATTATCAAAAACAACTTTGCT; Microsynth, Balgach, Switzerland).

### 2.8. Chromatin Immunoprecipitation Assay (ChIP)

ChIP analyses were done with the Millipore Magna ChIP Kit (Millipore, Darmstadt, Germany) as described previously [24,28]. To measure FOXO3 binding to the DEPP promoter (DEPP-forward: CTGCTCCTAGGAGAGACACACCCTG, DEPP-reverse: CTGCTACGTTTGCTGTGCTTAGTGC), real time RT-PCR analyses were performed.

### 2.9. Luciferase Activity Assay

To quantify FOXO3 binding to the DEPP, BIM, MMP-9, MMP-13 and LUM promoters, SH-EP/FOXO3 cells were transiently transfected using the LUC–promoter–reporter constructs specific to the DEPP promoter [28], the BIM promoter (provided by A. Villunger) [31], the MMP-9/MMP-13 promoters (provided by P. Storz) [4], or the LUM promoter purchased from Switchgear Genomics (Menlo Park, CA, USA), using the JetPrime^®^ Reagent (Polyplus, Berkeley, CA, USA) according to the manufacturer’s instructions. SH-EP/FOXO3 cells were treated with 4OHT alone, or in combination with RPG, for eight hours. Luciferase activity of the DEPP, BIM, MMP-9, and MMP-13 promoter constructs was measured with the Luciferase Assay System kit (Promega, Madison, WI, USA) and the LUM-specific luciferase reporter plasmid with the LightSwitch™ Luciferase Assay Kit (Switchgear Genomics, Menlo Park, CA, USA) according to the manufacturer’s instructions, as described [24].

### 2.10. Quantitative RT-PCR Analyses

RT-PCR analyses were performed to measure DEPP, BIM, and LUM mRNA expression, as described previously [32], using DEPP- (forward: ACTGTCCCTGCT CATCCATTCTC and reverse: AGTCATCCAGGCTAGGAGAGGG), BIM- (forward: AGCACCCATGAGTTGTGACAAATC and reverse: CGTTAAACTCGTCTCCAATACGC), LUM- (forward: TGAGCTGGATCTGTCCTATAA and reverse: ATCTTGCAGAAGCTCTTTATG), and GAPDH-specific oligonucleotides (forward: TGTTCGTCATGGGTGTGAACC and reverse: GCAGTGATGGCATGGACTGTG) [24].

### 2.11. Immunoblotting

Immunoblotting was performed as described previously [32] using antibodies directed against DEPP (Proteintech, Rosemont, IL, USA), BIM, NOXA, LUM, and GAPDH (Cell Signaling, Danvers, MA, USA). 

### 2.12. Determination of Cell Death by Flow Cytometry

Cell death induction was quantified as described [32]. 

### 2.13. Analysis of Cell Proliferation

The proportion of proliferating cells was analyzed using the BrdU cell proliferation ELISA kit (Abcam, Cambridge, UK) in a Benchmark Microplate Reader (BioRad Laboratories, Munich, Germany) according to the manufacturer’s instructions. 

### 2.14. Proteomics with Two-Dimensional Difference Gel Electrophoresis and Mass Spectrometry Analysis (2D-DIGE/MS)

Preparation of protein extracts of SH-EP/FOXO3 cells incubated with 75 nM 4OHT for 8 and 16 h was done as described previously [32]. Two-dimensional difference gel electrophoresis (2D DIGE) was used for separation of the proteins [33,34]. The protein expression was compared between untreated and treated cells. The gels were analyzed with the Typhoon 9410 imager (Amersham Biosciences, GE Healthcare) and the DeCyder 2D 6.5 software (GE Healthcare) using the biological variation analysis module to compare the spot ratios. Spots with a difference in the spot volume ≥1.5 with a *t*-test confidence ≥95% (*p* ≤ 0.05) between untreated und treated protein samples were excised with the Ettan Spot Picker and analyzed by mass spectrometry. 

### 2.15. Gelatin Zymography

To analyze MMP-13 enzymatic activity, IMR32/FOXO3 cells were pre-treated with 50 nM 4OHT and 50 µM RPG for eight hours. Subsequently, the cells were incubated with 50 nM 4OHT and 50 µM RPG in serum-free media for another 16 h before the supernatants were collected. A total of 450 µL of each supernatant was concentrated using Amicon^®^ Ultra centrifugal filters (Merck, Darmstadt, Germany) according to the manufacturer’s instructions. A total of 15 µL of each concentrated sample was subjected to gelatin zymography using 8% gelatin gels. After renaturation (2.5% Triton X-100 for one hour) and incubation at 37 °C for 22 h (1 M Tris-HCl pH 7.4, 1 M CaCl_2_, 5 M NaCl, 10% NaN_3_), the gels were stained with Coomassie blue. Subsequently, the gels were scanned and subjected to densitometric image analyses using the ImageJ 1.48 software.

### 2.16. 2D Migration Assay

To analyze the cellular 2D migration capabilities the two-well silicone inserts with a defined cell-free gap (ibidi, Martinsried, Germany) were pasted into 6-well plates. A total of 2 × 10^5^ SK-N-SH/FOXO3-shCTR, SK-N-SH/FOXO3-shLUM, IMR32/FOXO3-shCTR, or IMR32/FOXO3-shLUM cells were seeded into both spots of the culture-inserts to obtain a confluent layer within 24 h. After pre-incubation with 4OHT/RPG for 16 h, culture-inserts were removed by using sterile tweezers. Representative images were collected after 24 or 30 h of migration with the DMi8-inverted microscope (Leica, Wetzlar, Germany) and processed with the LAS X1.1.0 software (Leica, Wetzlar, Germany). The “open image area” was analyzed with the “T-scratch” software [35].

### 2.17. Microtissue Culture and 3D Spheroid Migration Assay

The generation of 3D spheroids was done with the GravityPLUS™ microtissue culture system (InSphero AG, Zürich, Switzerland) as described previously [24]. For the generation of spheroids of SK-N-SH/FOXO3-shCTR and SK-N-SH/FOXO3-shLUM cells, 2 × 10^4^ cells were seeded in 40 μL drops into the hanging drop plates and grown for 96 h. Spheroid size was monitored regularly by live-cell microscopy. For 3D migration analyses, the generated spheroids were subjected to the round-bottom 96-well plates coated with the ECM protein collagen I and treated with 4OHT/RPG. Representative images were collected after 24 h of migration with the DMi8 inverted microscope (Leica, Wetzlar, Germany) and processed with the LAS X1.1.0 software (Leica, Wetzlar, Germany). The area of migrating cells was calculated and outlined with the ImageJ 1.48 software.

### 2.18. Statistics

The statistical significance of differences between controls and treated cells was assessed using Student’s unpaired *t*-test unless stated otherwise.

## 3. Results

### 3.1. The FDA-Approved Compound RPG Displaces the IRE-FAM Oligonucleotide from the FOXO3-DBD

**Figure 1:**
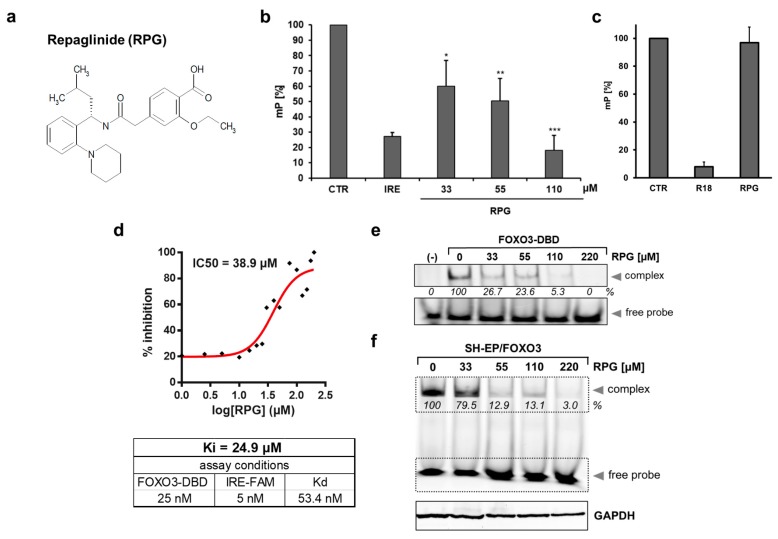
Displacement of the IRE-FAM oligonucleotide from the recombinant FOXO3-DBD protein by repaglinide (RPG). (**a**) Chemical structure of RPG. (**b**) Fluorescence polarization assay (FPA) analyses of the RPG interaction with the FOXO3-DBD protein (20 nM) using 5 nM IRE-FAM oligonucleotide in combination with 0.5 µM unlabelled IRE oligonucleotide, or with indicated concentrations of RPG. Shown are means ± s.e.m. of three independent experiments. * *p* < 0.05, ** *p* < 0.025, and *** *p* < 0.01 compared to the negative control (CTR). (**c**) FPA analyses of the RPG interaction with the R18 peptide/14-3-3 sigma protein (**d**) The IC_50_-value of RPG was measured by FPA using 25 nM FOXO3-DBD protein and 5 nM IRE-FAM oligonucleotide and calculated by nonlinear least-square analysis. Calculation of the Ki-value of RPG was performed by the equation of Nikolovska-Coleska. (**e**) By FAM-EMSA, the interaction of increasing concentrations of RPG with the FOXO3-DBD protein (1 µM and 100 nM fluorescence-labelled FoxP3 oligonucleotide) was analyzed. In the sample marked with (−), no FOXO3-DBD protein was added. The untreated control was set as 100%. (**f**) FAM-EMSA of SH-EP/FOXO3 extracts treated with 50 nM 4OHT in combination with indicated concentrations of RPG for four hours. By immunoblot analysis of GAPDH, equal loading of cellular protein extracts was ensured. Densitometric analysis was done with the ImageJ 1.48 software. The control (50 nM 4OHT) was set as 100%.

By an FPA-based screen using the Prestwick Chemical Library^®^ of 1120 approved drugs, we identified the small molecular weight molecules CBX and RPG as FOXO3 modulatory compounds [24]. Both substances bind to the recombinant GST-His purified FOXO3-DBD protein with a cut-off <31% mP-value [24]. Here, we verified the binding of RPG (Figure 1a) to the FOXO3-DBD and investigated the efficacy of RPG in silencing the transcriptional activity of FOXO3 in NB cells.

RPG dose-dependently interacted with the recombinant FOXO3-DBD protein, as determined by an FPA experiment using the fluorophore-labeled oligonucleotide containing the insulin response element (IRE). A total of 33 µM of RPG was sufficient to significantly suppress the interaction of the IRE-FAM oligonucleotide with the FOXO3-DBD protein. At a concentration of 110 µM, RPG quenched the mP-value to the same extent as a 100-fold (0.5 µM) higher concentration of the unlabeled IRE oligonucleotide (IRE, Figure 1b). The interaction of RPG with the FOXO3-DBD was validated using an FPA that determines the interaction of the 14-3-3 sigma protein with the R18 peptide [36]. At a concentration of 220 µM, RPG did not displace the R18 peptide from the 14-3-3 sigma protein (Figure 1c), which indicates that RPG specifically binds to the FOXO3-DBD protein. To measure the binding properties between RPG and the FOXO3-DBD protein, we determined the IC_50_ of RPG using 25 nM FOXO3-DBD protein and 5 nM FAM-labeled oligonucleotide. RPG was titrated with increasing concentrations (0–200 µM) and the IC_50_-value of 38.9 µM was calculated by nonlinear least-square analysis using the GraphPad Prism software. The binding affinity (Ki)-value of RPG was assessed by the equation of Nikolovska-Coleska [30] based on the measured IC_50_-values, the K-value of the protein/oligonucleotide complex (FOXO3-DBD/IRE-FAM oligonucleotide [24]), the concentration of the FOXO3-DBD protein (25 nM), and the IRE-FAM oligonucleotide (5 nM) used in the assay. We calculated a Ki-value of 24.9 µM for RPG with respect to our assay conditions (Figure 1d).

The interaction of RPG and the FOXO3-DBD protein was further assessed by FAM-EMSA using the FoxP3 oligonucleotide [37,38] that binds with high affinity to the FOXO3-DBD protein and forms a detectable shift band (Figure 1e). Incubation of the FOXO3-DBD protein/FoxP3-FAM oligonucleotide complex with increasing concentrations of RPG resulted in a dose-dependent loss of the band shift (Figure 1e). The specificity of the FOXO3-DBD protein/FoxP3-FAM oligonucleotide complex formation was validated previously by the use of mutated FoxP3 oligonucleotides [24]. To address whether RPG binds to FOXO3 in vitro, SH-EP/FOXO3 cells transgenic for the expression of a PKB-phosphorylation-independent, 4OHT-inducible FOXO3(A3) estrogen receptor ligand-binding domain (ERtm) plasmid [27] were incubated with 50 nM 4OHT to activate the FOXO3(A3)ERtm construct with different concentrations of RPG for four hours, and an FAM-EMSA was conducted. At a concentration of 55 µM, RPG inhibited the formation of the FoxP3-FAM oligonucleotide/FOXO3 complex. To ensure loading of the same protein concentration, GAPDH expression was analyzed by immunoblot (Figure 1f). In summary, these data demonstrate that RPG interacts with the FOXO3-DBD in NB cells.

### 3.2. RPG Silences the Transcriptional Activity of FOXO3 in Neuronal Cells

**Figure 2:**
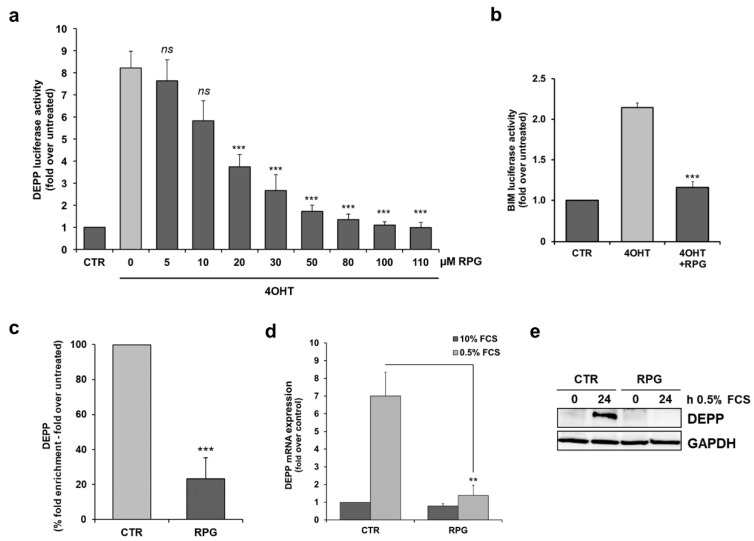
RPG inhibits the transcriptional activity of FOXO3 in NB cells. (**a**) A luciferase activity assay of SH-EP/FOXO3 cells (20 nM 4OHT), transfected with the DEPP-specific promoter plasmid and treated with the indicated concentrations of RPG for eight hours, was performed. The induction of the luciferase signal was calculated as fold over untreated controls. Shown are mean values ± s.e.m. of three independent experiments; *** *p <* 0.01 compared to 4OHT-treated cells. (**b**) A luciferase activity assay of SH-EP/FOXO3 cells transfected with a BIM-specific promoter reporter plasmid and treated with 50 nM 4OHT alone or in combination with 50 µM RPG for eight hours was done. Shown are the mean values ± s.e.m. of three independent experiments; *** *p <* 0.01 compared to 4OHT-treated cells. (**c**) SH-EP/FOXO3 cells were treated with 100 nM 4OHT alone or in combination with 30 µM RPG for three hours and ChIP analyses were performed. FOXO3 binding to the DEPP promoter was analyzed by quantitative RT-PCR analyses. Shown are the mean values ± s.e.m. of three independent experiments. *** *p* < 0.01 compared to the 4OHT-treated control (%). (**d**,**e**) The effect of RPG on FOXO3-mediated DEPP expression was quantified by quantitative RT-PCR (**d**) and by immunoblot analyses (**e**) in serum starved SH-EP cells (0.5% FCS) treated with 30 µM RPG for 24 h. Shown are the mean ± s.e.m. of three independent experiments. ** *p* < 0.025 compared to the serum starved control. GAPDH was used as loading control for immunoblot analyses.

In preceding studies, we characterized the pro-apoptotic BH3-only protein B-cell-lymphoma-gene-2-like-11 (BCL2L11/BIM) and the decidual protein induced by progesterone (DEPP/DEPP1/C10orf10) as transcriptional target genes of FOXO3 [27,28]. Therefore, DEPP- and BIM-specific luciferase promoter activity was analyzed in SH-EP/FOXO3 cells to investigate whether RPG is capable of silencing the transcriptional activity of FOXO3. In these cells, induction of the ectopically expressed FOXO3 transgene by 4OHT induced the luciferase activity of the DEPP promoter 8.2-fold (Figure 2a), and of the BIM promoter 2.2-fold (Figure 2b), compared to untreated cells. The FOXO3-induced DEPP promoter activity was repressed by RPG in a dose-dependent manner. A total of 20 µM RPG was sufficient to significantly (*p* < 0.01) inhibit the FOXO3-mediated DEPP promoter activity, and 50–80 µM RPG completely abrogated binding of FOXO3 to the DEPP promoter (Figure 2a). In line with this, RPG also efficiently repressed the binding of FOXO3 to the BIM promoter at a concentration of 50 µM (Figure 2b). ChIP analyses revealed that incubation with 30 µM RPG abolishes FOXO3 binding to the DEPP promoter in SH-EP/FOXO3 cells treated with 4OHT (Figure 2c). Of note, the needed RPG concentrations to silence the transcriptional activity of FOXO3 are in accordance with the concentrations investigated in the FAM-EMSA experiments (Figure 1e,f). As endogenous FOXO3 regulates the expression of DEPP during growth factor withdrawal [32], SH-EP cells were cultivated under serum starvation conditions (0.5% FCS) in the absence and presence of RPG. DEPP mRNA and protein expression regulated by endogenous FOXO3 was completely inhibited by RPG (Figure 2d,e). These results indicate that RPG silences the transcriptional target gene regulation of FOXO3 in neuronal tumor cells. 

### 3.3. RPG Represses FOXO3-Mediated NOXA, BIM, and DEPP Regulation and Associated Cell Death in Stroma-Like, Low-Stage SH-EP Cells

**Figure 3:**
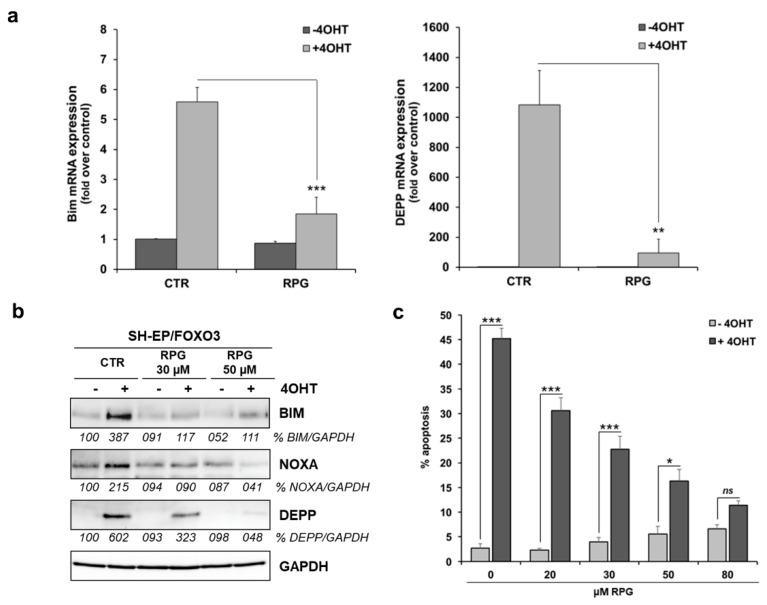
RPG represses FOXO3-triggered cell death in low-stage SH-EP cells. (**a**) Quantitative RT-PCR analyses of BIM and DEPP expression in SH-EP/FOXO3 cells treated with 20 nM 4OHT in combination with 30 µM RPG for six hours. Shown are means ± s.e.m. of three independent experiments. ** *p* < 0.025, *** *p* < 0.01 compared to the corresponding control. (**b**) Immunoblot analyses of SH-EP/FOXO3 cells treated with 50 nM 4OHT, and with indicated concentrations of RPG for eight hours, were performed to detect BIM, NOXA, and DEPP expression. GAPDH served as a loading control. Densitometric analyses were done with the ImageJ 1.48 software. Untreated cells were set as 100%. (**c**) PI-FACS analyses of SH-EP/FOXO3 cells treated with 20 nM 4OHT and with indicated concentrations of RPG for 48 h were performed to detect apoptotic cells. Shown are the mean values ± s.e.m. of three independent experiments. * *p* < 0.05, *** *p* < 0.01 compared to the corresponding control (one-way ANOVA).

In our previous studies we found that FOXO3 induces cell death in the low-stage SH-EP/FOXO3 cells via up-regulation of phorbol-12-myristate-13-acetate-induced-protein-1 (PMAIP1/NOXA), BIM, and DEPP [27,28,32]. As demonstrated by quantitative RT-PCR analyses, 30 µM RPG significantly repressed FOXO3-triggered expression of both BIM and DEPP in SH-EP/FOXO3 cells treated with 4OHT (Figure 3a). In concordance, RPG incubation dose dependently inhibited FOXO3-mediated BIM, DEPP, as well as NOXA protein expression (Figure 3b). Importantly, FOXO3-triggered apoptotic cell death was also diminished by RPG-treatment in a dose-dependent manner (Figure 3c) in SH-EP/FOXO3 cells. A total of 80 µM RPG repressed the FOXO3-triggered apoptosis rate to <15% apoptotic cells (Figure 3c). These results demonstrate that silencing of FOXO3 by RPG efficiently abrogates the FOXO3-triggered cell death program in “FOXO3-sensitive” SH-EP/FOXO3 cells.

### 3.4. RPG Silences FOXO3-Triggered MMP-9 and MMP-13 Promoter Activity

FOXO3 promotes cell invasion through the induction of the matrix metalloproteinases MMP-9 and MMP-13 in breast cancer cells [4]. FOXO3-dependent MMP activity and elevated invasion/migration capacity has also been reported in glioma [15], in gastric cancer [16], and in colorectal cancer [3]. Hence, we investigated the impact of FOXO3 on MMP-9 and MMP-13 promoter activity in the aggressive neuronal tumor cell lines IMR32 [39] and SK-N-SH [40]. IMR32 cells show a deletion of the short arm of chromosome 1 (1p) and a *MYCN* amplification in double minute chromosomes, whereas the SK-N-SH cells represent a stage 4 tumor. *MYCN* amplification and a 1p deletion are markers for aggressive advanced stage NB tumors [1]. Both cell lines were retrovirally infected with the 4OHT-inducible FOXO3(A3)-ERtm plasmid [29] (Figure S1).

**Figure 4:**
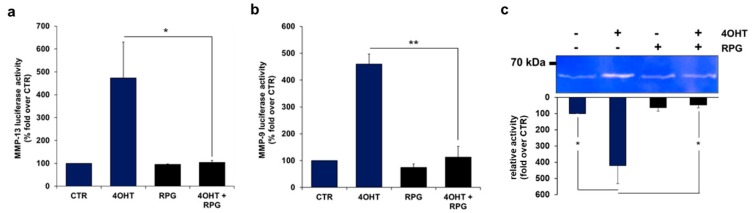
RPG silences FOXO3-triggered MMP-9 and MMP-13 promoter activity. (**a**) The MMP-13 promoter reporter plasmid was transfected into IMR32/FOXO3 cells. Twenty-four hours after transfection, IMR32/FOXO3 cells were treated with 50 nM 4OHT and 50 µM RPG for eight hours. A luciferase assay was performed to analyze the direct binding of FOXO3 to the MMP-13 promoter. The induction of the luciferase signal was determined as fold over untreated controls. Shown are the mean values ± s.e.m. of three independent experiments; * *p* < 0.05. (**b**) The MMP-9 promoter reporter plasmid was transfected into IMR32/FOXO3 cells which were treated with 50 nM 4OHT and 50 µM RPG for eight hours and a luciferase assay was performed. The induction of the luciferase signal was determined as fold over untreated controls. Shown are the mean values ± s.e.m. of three independent experiments; ** *p* < 0.025. (**c**) Gelatine zymography was performed to analyze the MMP-13 enzymatic activity. Shown is a representative zymogram demonstrating MMP-13 (61 kDa) activity in IMR32/FOXO3 cells incubated with 50 nM 4OHT and 50 µM RPG for 24 h. Densitometric analyses of MMP-13 activity were done with the ImageJ 1.48 software. Untreated cells were set as 100%. The increase in enzymatic activity was calculated as fold over untreated controls. Shown are the mean values ± s.e.m. of three independent experiments; * *p* < 0.05 compared to corresponding controls.

As demonstrated by luciferase promoter reporter analyses, ectopic activation of FOXO3 elevated the MMP-13 promoter activity 4.7-fold (Figure 4a), and the MMP-9 promoter activity 4.6-fold in IMR32/FOXO3 cells (Figure 4b), respectively. Notably, treatment with 50 µM RPG efficiently repressed FOXO3-mediated MMP-9 and MMP-13 promoter activation in these cells (Figure 4a,b).

By gelatine zymography analysis, we determined the enzymatic activity of MMP-13 in IMR32/FOXO3 cells. Activation of FOXO3 by 4OHT markedly induced the band of activity detected at 61 kDa, which is consistent with the active form of MMP-13 (Figure 4c). In line with the MMP-13 promoter activity experiment (Figure 4a), the FOXO3-mediated increase in the MMP-13 enzymatic activity was efficiently repressed by RPG treatment (Figure 4c). In summary, these results show that the transcription factor FOXO3 binds to the MMP-9 and MMP-13 promoters and elevates the MMP-13 enzymatic activity in neuronal tumor cells. Importantly, RPG efficiently silences the FOXO3-triggered regulation of these MMPs. 

### 3.5. RPG Inhibits the FOXO3-Mediated Regulation of LUM in NB Cells

**Figure 5:**
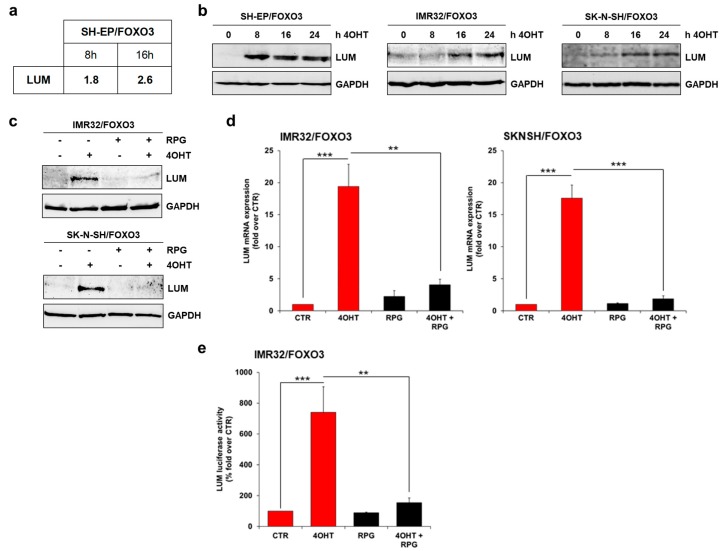
FOXO3 induces lumican (LUM) expression in NB cells. (**a**) LUM was identified as FOXO3-regulated protein in SH-EP/FOXO3 cells. SH-EP/FOXO3 cells were treated with 75 nM 4OHT for 8 and 16 h and subjected to 2D-DIGE/MS analyses. Shown are the fold-increases in LUM expression between untreated and 4OHT treated samples. (**b**) Immunoblot analyses of LUM expression in SH-EP/FOXO3, IMR32/FOXO3, and SK-N-SH/FOXO3 cells treated with 50 nM 4OHT to activate FOXO3 for the indicated time. GAPDH served as a loading control. (**c**) Immunoblot analyses of LUM expression in IMR32/FOXO3 cells treated with 50 nM 4OHT and 50 µM RPG, and in SK-N-SH/FOXO3 cells treated with 50 nM 4OHT and 80 µM RPG for 24 h, respectively. GAPDH served as a loading control. (**d**) Quantitative RT-PCR analyses of LUM expression in IMR32/FOXO3 and SK-N-SH/FOXO3 cells. IMR32/FOXO3 cells were preincubated with 50 µM RPG and SK-N-SH/FOXO3 cells with 80 µM RPG for one hour before incubation with 10 nM 4OHT for an additional six hours. Shown are the means ± s.e.m. of three independent experiments. ** *p* < 0.025, *** *p* < 0.01. (**e**) The LUM promoter reporter plasmid was transfected into IMR32/FOXO3 cells. 24 h after transfection the cells were treated with 50 nM 4OHT and 50 µM RPG for eight hours. A luciferase assay was performed to analyze direct binding of FOXO3 to the LUM promoter. The increase in the luciferase signal was calculated as fold over untreated controls. Shown are mean values ± s.e.m. of three independent experiments; ** *p* < 0.025; *** *p* < 0.01.

A proteomics approach by two-dimensional difference gel electrophoresis combined with mass spectrometry analysis (2D-DIGE/MS) identified the proteoglycan LUM as a potential FOXO3-regulated gene in SH-EP/FOXO3 cells. Activation of FOXO3 by 4OHT resulted in a time-dependent induction of LUM expression in these cells (Figure 5a). LUM has been reported to correlate with an advanced stage of pancreatic cancer [41] and with migration and invasive potential in gastric [19,20], colon [21] and bladder [22] cancer. In NB samples derived from primary tumors of untreated patients (“Tumor Neuroblastoma public; Versteeg; 88; MAS5.0; u133p2” dataset [42]), high LUM expression is associated with a reduced overall survival probability, as estimated by Kaplan–Meier analysis performed with the R2 bioinformatic platform (http://r2.amc.nl; Figure S2).

In line with the 2D-DIGE/MS experiment, we found LUM protein expression up-regulated due to ectopic FOXO3 activation in SH-EP/FOXO3, IMR32/FOXO3, and SK-N-SH/FOXO3 cells (Figure 5b). In concordance, LUM induction upon FOXO3 activation was also detectable on the mRNA level in IMR32/FOXO3 and SK-N-SH/FOXO3 cells (Figure 5d). 

As demonstrated by immunoblot, as well as by quantitative RT-PCR analyses, FOXO3-regulated LUM expression was efficiently abrogated by FOXO3 inhibition with 50 µM RPG in IMR32/FOXO3 cells and with 80 µM RPG in SK-N-SH/FOXO3 cells (Figure 5c,d). To verify the observed effect of RPG on FOXO3-triggered LUM expression, we performed LUM-specific luciferase promoter activity analyses. Activation of FOXO3 by 4OHT elevated the LUM luciferase activity 7-fold in IMR32/FOXO3 cells (Figure 5e). Consistent with the immunoblot (Figure 5c) and the RT-PCR analyses (Figure 5d), RPG markedly repressed the binding of FOXO3 to the LUM promoter at a concentration of 50 µM in IMR32/FOXO3 cells (Figure 5e). Together, these findings demonstrate that FOXO3 induces the expression of LUM, and that RPG efficiently represses FOXO3-mediated LUM induction in neuronal tumor cells.

### 3.6. RPG Represses the FOXO3/LUM-Triggered 2D and 3D Migration in NB Cells

**Figure 6:**
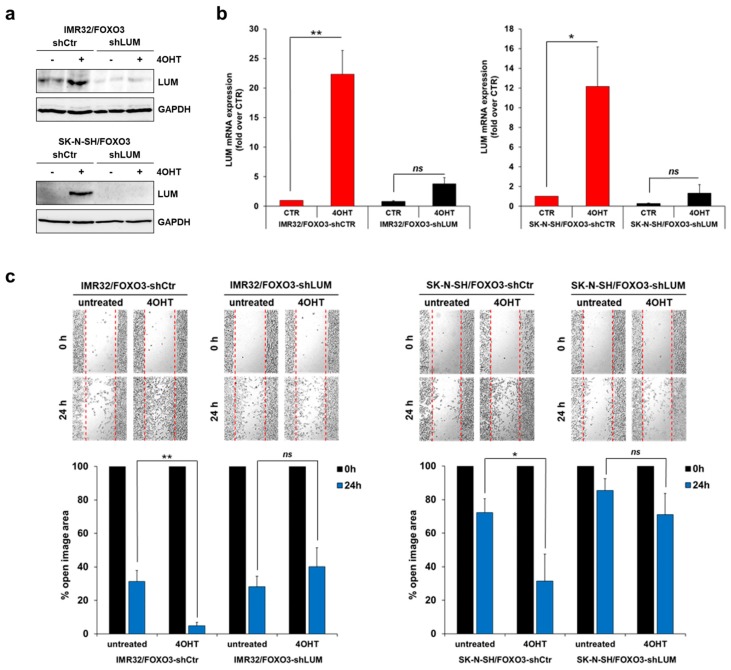
FOXO3-mediated migration depends on LUM expression in NB. (**a**) IMR32/FOXO3-shCTR, IMR32/FOXO3-shLUM, SK-N-SH/FOXO3-shCTR, and SK-N-SH/FOXO3-shLUM cells were treated with 50 nM 4OHT for 24 h and subjected to immunoblot analyses to detect LUM expression. GAPDH served as a loading control. (**b**) Quantitative RT-PCR analyses of LUM expression in IMR32/FOXO3-shCTR, IMR32/FOXO3-shLUM, SK-N-SH/FOXO3-shCTR, and SK-N-SH/FOXO3-shLUM cells treated with 10 nM 4OHT for six hours. Shown are the mean ± s.e.m. of three independent experiments. * *p* < 0.05, ** *p* < 0.025 compared to the corresponding control. (**c**) The 2D migration assay was performed with SK-N-SH/FOXO3-shCTR and SK-N-SH/FOXO3-shLUM cells (left panel), as well as with IMR32/FOXO3-shCTR and IMR32/FOXO3-shLUM cells (right panel), seeded in both spots of the culture-insert to obtain a confluent layer within 24 h. After pre-incubation of the cells with 50 nM 4OHT for 16 h, the culture-inserts were removed. Representative images were collected starting immediately after culture-insert removal and after 24 h. The “open image area” was calculated with the “T-scratch” software. Shown are the mean values ± s.e.m. of three independent experiments; * *p* < 0.05, ** *p* < 0.025.

To assess the impact of FOXO3 and LUM on the migration capacity of aggressive NB cells in vitro, we performed 2D and 3D migration analyses. For that purpose, we performed stable LUM-knockdown by expression of LUM-specific shRNA in IMR32/FOXO3 and SK-N-SH/FOXO3 cells, respectively. FOXO3-mediated induction of LUM expression in IMR32/FOXO3 and SK-N-SH/FOXO3 cells was markedly repressed by the stable expression of LUM-specific shRNA on the protein level (Figure 6a) and on the mRNA level (Figure 6b). The 2D “wound healing” assay revealed a significant FOXO3-triggered increase in cellular migration in SK-N-SH/FOXO3-shCTR and in IMR32/FOXO3-shCTR cells treated with 4OHT (Figure 6c). Stable LUM-knockdown in SK-N-SH/FOXO3-shLUM, as well as in IMR32/FOXO3-shLUM cells, abrogated the observed increase in cellular migration, indicating that FOXO3-mediated 2D migration depends on LUM expression in NB cells (Figure 6c). Of note, we reported that FOXO3 induces cell death in low-stage “FOXO3-sensitive” SH-EP/FOXO3 cells (Figure 3c) [27,28,32]. Importantly, FOXO3/LUM activation did not affect cell viability/cell death in the aggressive SK-N-SH/FOXO3 and IMR32/FOXO3 cells (Figure S3). In these cells, FOXO3 repressed cell proliferation, as demonstrated by the BrdU incorporation analyses (Figure S4), indicating that FOXO3 triggered migration is executed independently of cellular proliferation.

**Figure 7:**
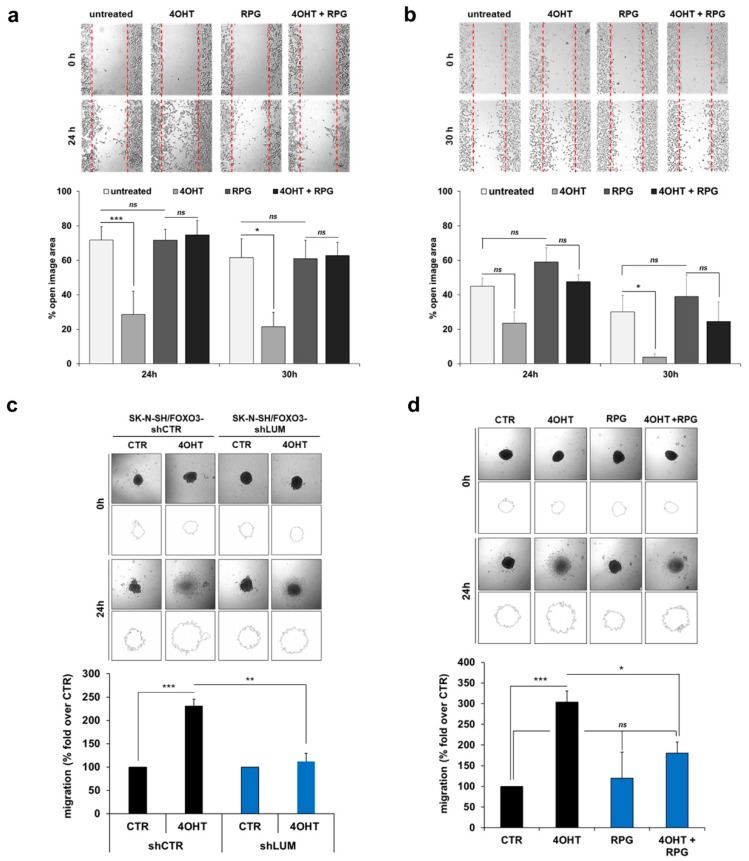
RPG treatment represses FOXO3-mediated 2D and 3D migration. (**a**) The 2D migration assay was performed with IMR32/FOXO3 cells seeded in both spots of the culture-insert to obtain a confluent layer within 24 h. After pre-incubation with 50 nM 4OHT and/or 30 µM RPG for 16 h the culture-inserts were removed and representative images were collected starting immediately after culture-insert removal, after 24 and 30 h. The “open image area” was calculated with the “T-scratch” software. Shown are the mean values ± s.e.m. of three independent experiments; * *p* < 0.05, *** *p* < 0.01 (one-way ANOVA). (**b**) The 2D migration assay was performed with SK-N-SH/FOXO3 cells seeded in both spots of the culture-insert to obtain a confluent layer within 24 h. After pre-incubation with 50 nM 4OHT and/or 80 µM RPG for 16 h the culture-inserts were removed and representative images were collected, starting immediately after culture-insert removal, and after 24 and 30 h. The “open image area” was calculated with the “T-scratch” software. Shown are the mean values ± s.e.m. of three independent experiments; * *p* < 0.05 (one-way ANOVA). (**c**) To analyze the 3D migration capacity, spheroids derived from SK-N-SH/FOXO3-shCTR and SK-N-SH/FOXO3-shLUM cells were transferred into plates coated with the ECM protein collagen I and subsequently treated with 100 nM 4OHT. Representative images were collected after 24 h of migration. The area of migrating cells was calculated and outlined with the ImageJ 1.48 software. Shown are the mean values ± s.e.m. of three independent experiments; ** *p* < 0.025, *** *p* < 0.01. (**d**) The 3D migration assay was performed with spheroids derived from SK-N-SH/FOXO3 cells in plates coated with the ECM protein collagen I. The spheroids were treated with 100 nM 4OHT and/or 80 µM RPG and representative images were collected after 24 h of migration. The area of migrating cells was calculated and outlined with the ImageJ 1.48 software. Shown are the mean values ± s.e.m. of three independent experiments; * *p* < 0.05, *** *p* < 0.01 (one-way ANOVA).

Consistent with the LUM-knockdown experiments, the FOXO3-triggered increase in cellular migration was efficiently abrogated by FOXO3 inhibition with 30 µM RPG in IMR32/FOXO3 cells (Figure 7a) and with 80 µM RPG in SK-N-SH/FOXO3 cells (Figure 7b), respectively. Notably, at the applied concentration, RPG affected neither cell proliferation (Figure S4) nor cell viability/cell death (Figure S5) in IMR32/FOXO3 and in SK-N-SH/FOXO3 cells.

Stimulated cell migration by FOXO3 activation was also visible in a 3D spheroid-based migration experiment. SK-N-SH cells were selected for this approach as they form spheroids with a compact uniform shape and consistent size. In line with the 2D “wound healing” assay, ectopic FOXO3 expression elevated the 3D migration of SK-N-SH/FOXO3 spheroids (Figure 7c,d). This increase in FOXO3-triggerd 3D migration was markedly repressed by LUM-knockdown in SK-N-SH/FOXO3-shLUM cells, indicating that LUM expression is also necessary to facilitate FOXO3-dependent cell migration in the 3D setting (Figure 7c). Consistently, FOXO3 inhibition by 80 µM RPG significantly lowered the 3D migration capacity in SK-N-SH/FOXO3 spheroids (Figure 7d).

In summary, these results indicate that FOXO3-mediated migration depends on LUM expression in neuronal tumor cells. Consistently, FOXO3 inhibition by RPG efficiently repressed the 2D and 3D migration of NB cells.

## 4. Discussion

The oncogenic properties of the transcription factor FOXO3 have been reported in different cancers [2,3,4,5,6,7,8,9,10]. In high-stage NB, FOXO3 promotes tumor angiogenesis in vivo [11] and chemoprotection in vitro [12]. In addition, several studies point out a supportive role of FOXO3 in facilitating and stimulating metastasis formation (reviewed in [13]), one of the biggest challenges in the treatment of aggressive NB [1]. Hence, the pharmacological inhibition of FOXO3 is of great interest for the treatment of NB and other FOXO3-dependent tumors. In the present study, we demonstrate for the first time that FOXO3 elevates the migratory capacity of NB cells via LUM expression and characterize the FDA-approved compound RPG as a novel FOXO3 inhibitor that efficiently represses the metastatic potential of neuronal cancer cells.

By a drug-repositioning strategy and screening of the Prestwick Chemical Library^®^, we recently identified the glycyrrhetinic acid derivative CBX as the first FDA-approved small molecular weight inhibitor of the transcription factor FOXO3 [24]. CBX interacts with the FOXO3-DBD, thereby silences its transcriptional activity, and overcomes FOXO3-mediated chemoprotection in aggressive NB [24]. Besides CBX, RPG (Figure 1a), a carbamoylbenzoic acid derivative related to the meglitinide class of insulin secretagogues [43], was also discovered as a putative FOXO3 inhibitory compound [24]. RPG represents an FDA-approved drug for the treatment of type 2 diabetes mellitus (DM) [43], that rapidly lowers blood glucose levels by stimulating the insulin release from the pancreas through inhibition of the K_ATP_ channel activity [44]. Of note, DM treatment by RPG might trigger beneficial side effects through FOXO3 inhibition, as a number of studies suggest that inactivation of FOXO proteins may foster cytoprotection in endothelial cells during DM [45,46], prevent insulin resistance through the inhibition of hepatic gluconeogenesis [47], and prevent retinal disease in murine models [48]. Regarding its potential use in cancer, RPG was identified as a putative new drug for the treatment of glioblastoma multiforme by a Connectivity Map screen. RPG inhibited the proliferation and migration of glioblastoma multiforme cells and in vivo experiments revealed that RPG prolonged the median survival time of mice bearing orthotopic glioma [49]. However, the detailed mechanism of how RPG affects cancer cells has not been described so far.

In the present study, we validated the interaction of RPG with FOXO3-DBD. RPG binds to the FOXO3-DBD with a binding affinity of 24.9 µM (Figure 1d) and in a dose-dependent manner, as demonstrated by FPA (Figure 1b) and EMSA (Figure 1e,f). We used the low-stage [50] “FOXO3-sensitive” SH-EP/FOXO3 cell line, that stably expresses a 4OHT-inducible, PKB-phosphorylation-independent FOXO3(A3)ERtm transgene [27], to initially analyze whether RPG is capable of silencing the FOXO3 transcriptional activity in vitro. RPG efficiently abrogated promoter binding of FOXO3 to its transcriptional target DEPP [28], as demonstrated by promoter reporter assay (Figure 2a) and by ChIP (Figure 2c). Furthermore, the binding of FOXO3 to the promoter of its target gene BIM [27] was suppressed by RPG incubation (Figure 2b). Notably, RPG efficiently abrogated DEPP expression triggered by starvation-induced endogenous FOXO3 [28] on protein and RNA levels (Figure 2d,e). RPG incubation inhibited the FOXO3-regulated expression of NOXA, BIM, and DEPP on RNA (Figure 3a) and protein levels (Figure 3b) and consequently inhibited FOXO3-dependent apoptosis in low-stage “FOXO3-sensitive” SH-EP/FOXO3 cells in a dose-dependent manner (Figure 3c). Collectively, our data indicate that RPG is capable of silencing the transcriptional activity of FOXO3 in neuronal tumor cells.

The metastatic potential of neuronal tumor cells contributes to a high relapse rate and a five year survival probability of less than 40% in aggressive NB [1]. FOXO3 promotes breast cancer cell invasion through the induction of MMP-9 and MMP-13 [4] and has further been associated with MMP-9 activity and elevated invasion capacity in glioma [15], and with increased cell migration and invasion in gastric [16] and colorectal cancer [3]. In concordance with the concept that FOXO3 positively regulates MMPs and increases cellular migration, we found that FOXO3 binds to the MMP-13 and the MMP-9 promoter in IMR32/FOXO3 cells. Importantly, RPG treatment efficiently repressed FOXO3 binding to both promoters (Figure 4a,b). In line with this, a gelatin zymography experiment revealed that the FOXO3-triggered increase in MMP-13 enzymatic activity was abrogated by RPG incubation (Figure 4c).

By a proteomics approach, we identified the SLRP LUM as a FOXO3-regulated target in NB (Figure 5a). The FOXO3-triggered LUM expression was visible on protein (Figure 5b) and on RNA levels (Figure 5d) in different NB cell lines. SLRPs are ubiquitous extracellular matrix (ECM) components involved in matrix structural organization and the regulation of cancer cell multiplication, angiogenesis, and migration. Although the impact of LUM expression on cancer progression seems to be tumor specific, its expression is strikingly upregulated in various tumor sites including lungs, stomach, colon, pancreas, and urinary bladder. In colon cancer cells, the overexpression of LUM leads to actin cytoskeletal remodeling and an increased migration capacity [21]. Hence, LUM expression contributes to migration and metastasis formation in a tumor-specific manner (reviewed in [51]). In NB, high LUM expression is associated with lower overall survival (Figure S2). Of note, Farace et al. reported that LUM expression is associated with the maintenance of a quiescent, drug-resistant, stem-cell-like phenotype in NB and glioblastoma cells [23]. However, the impact of LUM on neuronal tumor cell migration has not been investigated to date. Here, we demonstrate for the first time that FOXO3 activation triggers cellular migration in aggressive SK-N-SH/FOXO3 and IMR32/FOXO3 cells. Notably, stable LUM-knockdown abrogated FOXO3-mediated cellular 2D (Figure 6c) and 3D migration (Figure 7c), indicating that FOXO3-triggered cellular migration depends on LUM expression.

RPG inhibited FOXO3-mediated LUM expression on protein (Figure 5c) and RNA level (Figure 5d), as well as the binding of FOXO3 to the LUM promoter (Figure 5e). In concordance with the LUM-knockdown experiments (Figure 6c and Figure 7c), RPG abrogated the FOXO3-dependent increase in cellular migration in the 2D “wound healing” assay in SK-N-SH/FOXO3 (Figure 7a) and IMR32/FOXO3 cells (Figure 7b). FOXO3-silencing by RPG further repressed the FOXO3-triggered 3D migration in tumor spheroids derived from high-stage SK-N-SH/FOXO3 cells (Figure 7d).

## 5. Conclusions

In summary, we describe the FDA-approved small molecular weight compound RPG as putative inhibitor of the transcription factor FOXO3. RPG interferes with the FOXO3-DBD and thereby silences the transcriptional activity of FOXO3 in neuronal tumor cells. FOXO3 inhibition by RPG represses its binding to the LUM promoter and efficiently abrogates FOXO3/LUM-triggered cellular migration in a 2D and 3D setting. Hence, we propose that silencing the FOXO3/LUM axis by the FDA-approved compound RPG represents a promising starting point to develop novel therapies targeting the metastatic potential of aggressive NB.

## Supplementary Materials

The following are available online at https://www.mdpi.com/2073-4409/9/1/1/s1, Figure S1: Immunoblot analyses of endogenous/exogenous FOXO3 expression in SK-N-SH and SK-N-SH/FOXO3 cells, Figure S2: LUM expression is associated with a reduced overall survival probability in NB, Figure S3: FOXO3/LUM activation does not affect cell viability, Figure S4: Impact of FOXO3 activation and of RPG treatment on cell proliferation/BrdU incorporation, Figure S5: RPG does not affect cell viability.
