# Supplementary material for: Repaglinide Silences the FOXO3/Lumican Axis and Represses the Associated Metastatic Potential of Neuronal Cancer Cells
Source: Cells. 2019 Dec 18;9(1):1. doi: 10.3390/cells9010001 (PMC7017090; doi:10.3390/cells9010001)
Supplement: Supplementary file 1 [file cells-09-00001-s001.pdf]

Figure S1.

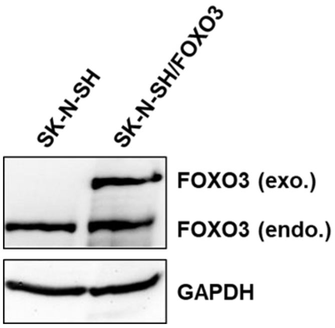

Figure S1. Immunoblot analyses of endogenous/exogenous FOXO3 expression in SK-N-SH and SK-N-SH/FOXO3 cells. GAPDH served as loading control.

Figure S2.

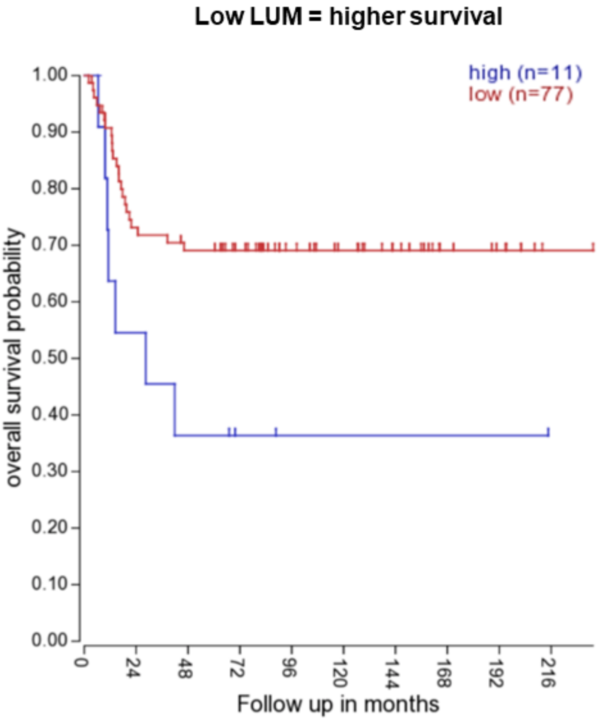

Figure S2. Kaplan scanning of the "Tumor Neuroblastoma public; Versteeg; 88; MAS5.0; u133p2" dataset using the R2 bioinformatic platform (http://r2.amc.nl). R2 calculates for each gene of interest the optimal cut-off expression level dividing the patients in a good and bad prognosis cohort. The Kaplan-Meier estimator indicated that LUM expression is associated with a reduced overall survival probability in NB (LUM: 201744_s_at; expression cutoff: 2525.6).

Figure S3.

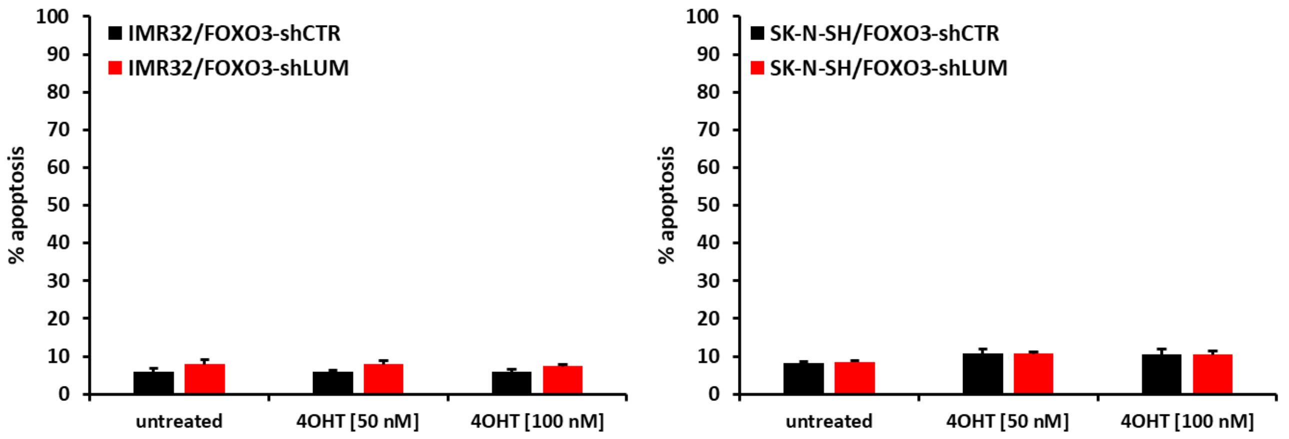

Figure S3. IMR32/FOXO3-shCTR and IMR32/FOXO3-shLUM, as well as SK-N-SH/FOXO3-shCTR and SK-N-SH/FOXO3-shLUM cells were treated with indicated concentrations of 4OHT for 24 hours. PI-FACS analyses were performed to detect apoptotic cells. Shown are mean values ± s.e.m. of three independent experiments.

Figure S4.

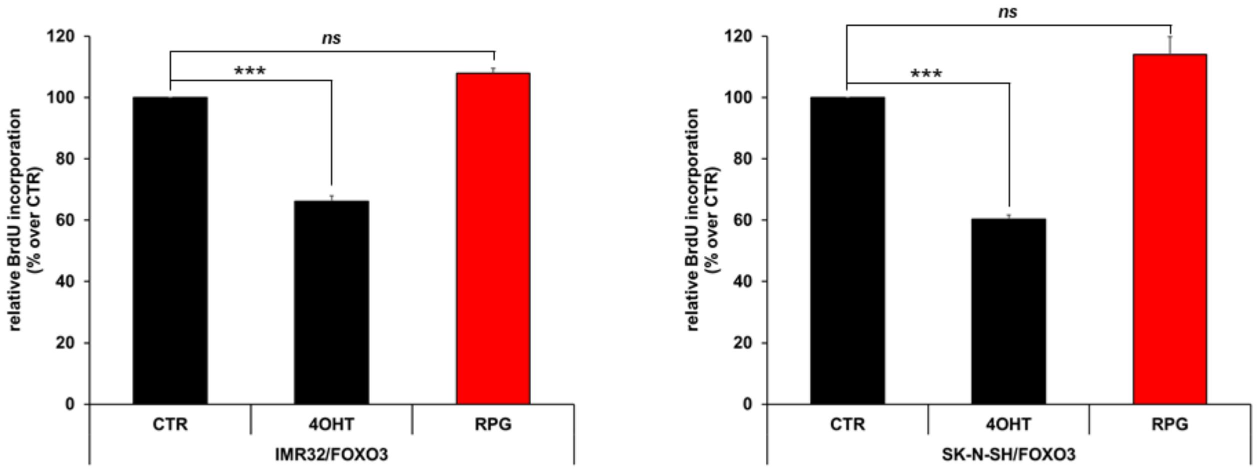

Figure S4. Analyses of the BrdU incorporation in IMR32/FOXO3 cells treated with 100 nM 4OHT and 30 µM RPG (left panel) and in SK-N-SH/FOXO3 cells treated with 100 nM 4OHT and 80 µM RPG (right panel) for 24 hours. Shown are mean values ± s.e.m. of three independent experiments. ***P < 0.01 compared to the untreated control.

Figure S5.

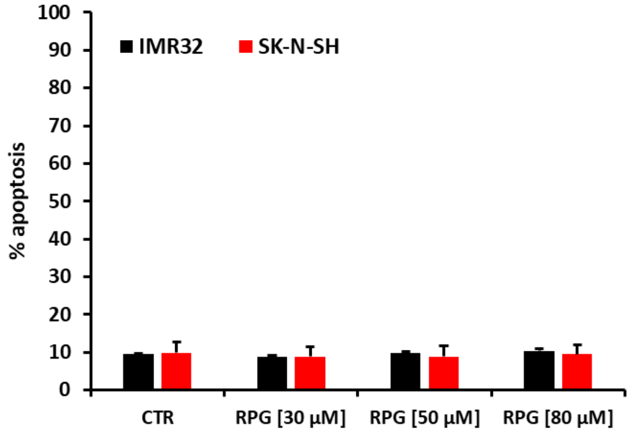

Figure S5. IMR32 and SK-N-SH cells were treated with indicated concentrations of RPG for 24 hours. PI-FACS analyses were performed to detect apoptotic cells. Shown are mean values ± s.e.m. of three independent experiments.
